# TDP‐43 as structure‐based biomarker in amyotrophic lateral sclerosis

**DOI:** 10.1002/acn3.51256

**Published:** 2020-12-02

**Authors:** Léon Beyer, René Günther, Jan Christoph Koch, Stephan Klebe, Tim Hagenacker, Paul Lingor, Anne‐Sophie Biesalski, Andreas Hermann, Andreas Nabers, Ralf Gold, Lars Tönges, Klaus Gerwert

**Affiliations:** ^1^ Center for Protein Diagnostics (ProDi) Ruhr University Bochum Bochum Germany; ^2^ Faculty of Biology and Biotechnology Department of Biophysics Ruhr University Bochum Bochum Germany; ^3^ Department of Neurology Technische Universität Dresden Dresden Germany; ^4^ German Center for Neurodegenerative Diseases (DZNE) Dresden Dresden Germany; ^5^ Department of Neurology University Medicine Göttingen Göttingen Germany; ^6^ Department of Neurology University Hospital Essen Essen Germany; ^7^ School of Medicine Klinikum rechts der Isar Department of Neurology Technical University of Munich Munich Germany; ^8^ Department of Neurology St. Josef‐Hospital Ruhr‐University Bochum Bochum Germany; ^9^ Translational Neurodegeneration Section “Albrecht‐Kossel” Department of Neurology University Medical Center Rostock University of Rostock Rostock Germany; ^10^ German Center for Neurodegenerative Diseases (DZNE) Rostock/Greifswald Rostock Germany

## Abstract

Pathologic alterations of Transactivation response DNA‐binding protein 43 kilo Dalton (TDP‐43) are a major hallmark of amyotrophic lateral sclerosis (ALS). In this pilot study, we analyzed the secondary structure distribution of TDP‐43 in cerebrospinal fluid of ALS patients (*n* = 36) compared to Parkinson´s disease patients (PD; *n* = 30) and further controls (Ctrl; *n* = 24) using the immuno‐infrared sensor technology. ALS patients could be discriminated from PD and Ctrl with a sensitivity/specificity of 89 %/77 % and 89 %/83 %, respectively. Our findings demonstrate that TDP‐43 misfolding measured by the immuno‐infrared sensor technology has the potential to serve as a biomarker candidate for ALS.

## Introduction

Amyotrophic lateral sclerosis (ALS) is a fatal neurodegenerative disease with rapid loss of motor function. It leads to necessity of artificial nutrition, ventilation or results in death within few years after diagnosis. To date, one of the key challenges in ALS diagnostics is to exclude other mimicking diseases, which are assessed based on clinical and electrophysiological parameters, while disease‐specific diagnostic and prognostic biomarkers are still lacking. Recently, neurofilament light chain and phosphorylated neurofilament heavy chain gained attention as potential diagnostic and prognostic biomarkers for ALS.^1^ However, the neurodegenerative demise in ALS is associated with elevated cerebrospinal fluid (CSF) and blood serum neurofilament levels, but this biomarker is not specifically correlated with ALS occurrence such as elevated levels of Tau in various types of dementia and brain injuries.^2^, ^3^ Transactivation response DNA‐binding protein 43 kDa (TDP‐43) cytoplasmic inclusions are the major neuropathological hallmark in sporadic and most genetic ALS^4^ cases and were found in most brain autopsies of ALS patients.^5^ So far, only a few studies evaluated the potential of TDP‐43 as a biomarker using enzyme‐linked immunosorbent assay (ELISA) or western blot analysis in CSF of ALS patients with heterogeneous results.^3^ Under pathological conditions, TDP‐43 is subjected to hyperphosphorylation, ubiquitination and N‐terminal truncation and demonstrates nonphysiological morphological and conformational alterations.^3^, ^5^ Standard immunoassays are methodically limited to analyze the TDP‐43 misfolding state, are prone for unspecific adsorption and lack spectral quality control which might lead to ineffective detection of the target protein. More importantly, techniques that merely analyze concentration or size of the protein do not provide information about structural aspects that trigger cytoplasmic inclusions. Therefore, a more detailed analysis of the molecular and structural pathology of TDP‐43 is needed. For this purpose, we applied the immuno‐infrared sensor technique which had been applied recently to detect the secondary structure distribution of Amyloid‐β and Tau in CSF and blood plasma of Alzheimer’s disease patients as structure biomarkers.^6^, ^7^, ^8^ This structure‐based biomarker methodology was validated as predictive marker in cohorts comprising different stages of Alzheimer´s disease, mainly early‐ or even pre‐symptomatic disease stages.^6^, ^7^, ^8^, ^9^, ^10^ The aim of this pilot study was to evaluate if TDP‐43 has potential to be used as a structure biomarker for ALS.

## Methods

### Patients

Between 2016 and 2018, CSF samples of 36 patients diagnosed with ALS according to the revised El Escorial criteria,^11^, ^12^ 30 Parkinson´s disease (PD) patients diagnosed according to the United Kingdom PD Society Brain Bank clinical diagnostic criteria (PMID 2841426) and 24 subjects suffering from further neurological diseases (Ctrl) (including two purine nucleoside phosphorylase, one normal pressure hydrocephalus, one essential tremor, five polyneuropathies, one dystonia and ataxia, one myasthenia gravis, seven Alzheimer’s disease, one myelitis, one epileptic seizure, one sarcoidosis, one multiple sclerosis, one complex partial seizures, one chronic inflammation) were collected at four academic centers for neurological diseases (Dresden, Essen, Göttingen, and Bochum) all following SOP procedures which are in accordance with current clinical GCP and research guidelines. For ALS patients, disease subtype at onset, body mass index (BMI), forced vital capacity, percentage of predicted forced vital capacity (adapted to age, sex, and body height for each patient), disease duration (time between the first symptom and the date of sample collection), ALS functional rating scale – revised score (ALSFRS‐R) and ALSFRS‐R slope, calculating the decline of ALSFRS‐R from symptom onset to the date of sample collection $\left(\frac{48‐\text{score}\;\text{at}\;\text{date}\;\text{of}\;\text{sample}\;\text{collection}}{\text{duration between symptom onset and date of sample collection}}\right)$ were collected (for details see Table 1). All subjects gave their informed consent and study approval was obtained by the local ethics committee and institutional review boards (Dresden EK393122012; Göttingen No.13/11/12; Essen No.18‐WBE‐058; Bochum No. 17‐6119).

**Table 1:** Demographic and clinical characteristics of the study population.

	ALS	PD	Ctrl	Mann–Whitney *U*‐test		Chi^2^
Number	36	30	24			
Ratio of female (%)	61	33	46			ALS versus PD (*P* = 0.02) ALS versus Ctrl (*P* = 0.24) PD versus Ctrl (*P* = 0.35)
Age (y)	66 ± 10	69 ± 13	65 ± 17	ALS versus PD (*P* = 0.81) ALS versus Ctrl (*P* = 0.54) PD versus Ctrl (*P* = 0.35)		
BMI	25.7 ± 3.9	—	—			
Disease onset	25 spinal/ 11 bulbar	—	—			
Disease duration (yr)	1.5 ± 1.4	—	—			
CSF‐to‐serum albumin quotient concentrations (Qalb) versus amide I maximum position				ALS (*P* = 0.81, *r *= −0.36) PD (*P* = 0.18, *r* = −0.25) Ctrl (*P* = 0.002, *r* = −0.62)		
ALSFRS‐R	38 ± 7 (Range: 16–47)	—	—			
ALSFRS‐R slope	0.92 ± 0.89 (Range: 0.06–3.48)	—	—			
Forced vital capacity (mL)	2350 ± 962 (Range: 360–4004)	—	—			
Percentage of predicted forced vital capacity (%)	69.3 ± 22.4 (Range: 12–111)	—	—			
TDP‐43 amide I maximum frequency mean (cm^−1^) + SD (cm^−1^)	1639.5 ± 2.6 (Range: 1634–1646)	1651.5 ± 7.7 (Range: 1637–1662)	1646 ± 5.5 (Range: 1639–1658)	ALS versus PD (*P* < 0.0001) ALS versus Ctrl (*P* < 0.0001) PD versus Ctrl (*P* = 0.20)		

Body mass index (BMI); Amyotrophic lateral sclerosis functional rating scale‐revised (ALSFRS‐R); cohort of patients with Amyotrophic lateral sclerosis (ALS); cohort of patients with Parkinson´s disease (PD); cohort of patients with other neurological diseases (Ctrl).

### Determination of the TDP‐43 misfolding state in CSF using the immuno‐infrared sensor

**Figure 1:**
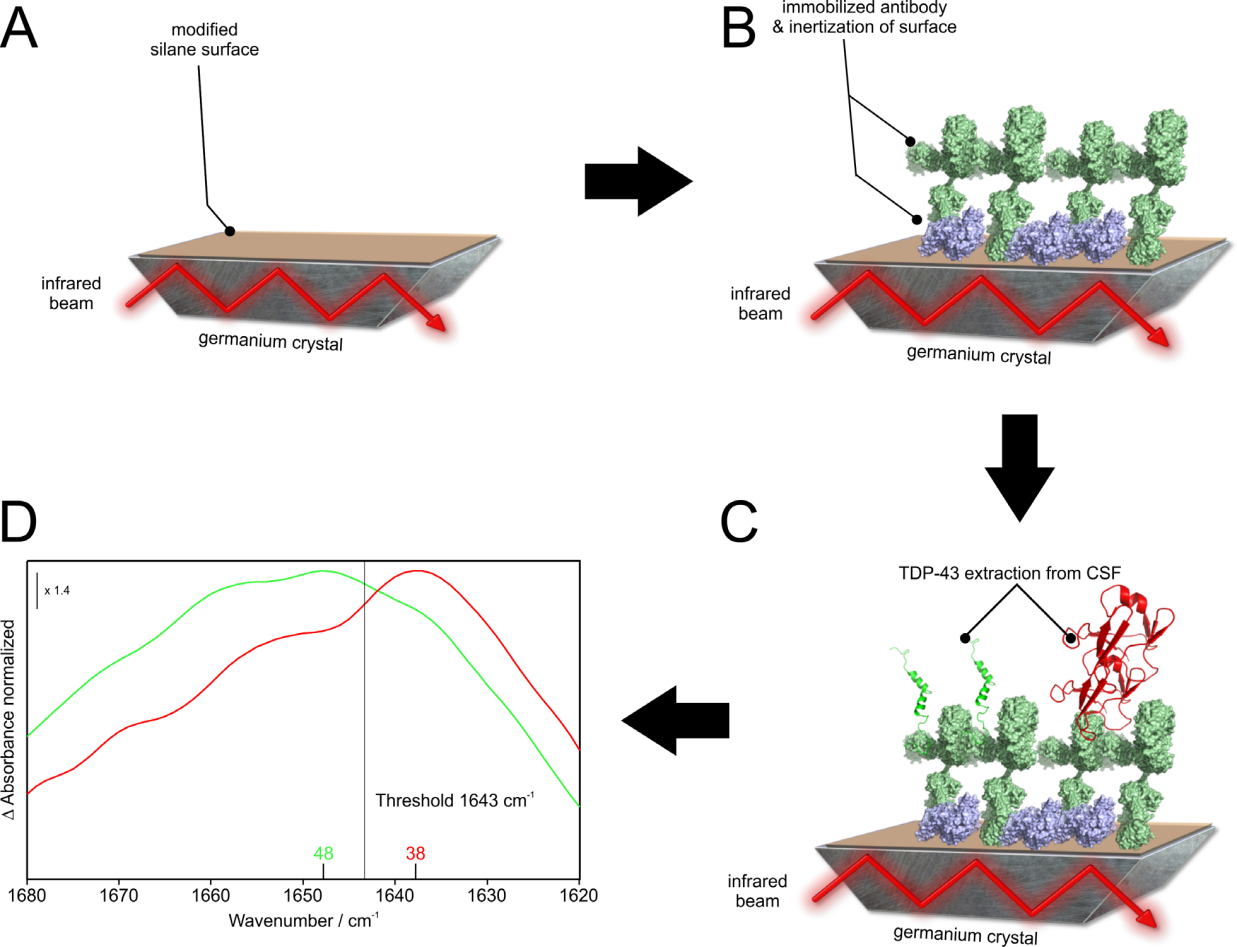
Schematic illustration of the immuno‐infrared sensor. Using oligoclonal capture antibodies, all TDP‐43 isoforms are extracted out of CSF. (A) First, a germanium crystal (grey) is chemically functionalized by using a self‐synthesized silane (brown). (B) In a second step the antibody (light green) is covalently attached to the functionalized silane sensor surface (brown) of a germanium crystal (grey). Afterwards, free binding sites on the surface are blocked by casein (purple). (C) The infrared beam is guided through the internal refection element and at the reflection points an evanescent wave measures the absorbance of the attached thin layer consisting of silane, casein, antibody, buffer, and TDP‐43 extracted out of CSF (green and red). (D) By performing a difference spectrum between background and bound TDP‐43 the secondary structure sensitive amide I band is revealed. The frequency of the absorbance maximum indicates the secondary structure distribution and misfolding state of TDP‐43. In this context, a maximum frequency of 1648 cm^−1^ (green) shows mainly *α*‐helical or disordered structures, whereas a maximum of 1638 cm^−1^ (red) indicates a signal of *β*‐sheet enriched pathological structures.

The immuno‐infrared sensor is an universal technology that provides a relative measure directly reflecting the secondary structure distribution of a biomarker in a biofluid. The samples are analyzed in a flow‐through system. As read‐out the so‐called amide I absorbance band is recorded, which reflect the secondary structure sensitive C=O stretching vibration of the protein backbone (Fig. 1). Pure monomeric – alpha‐helical or disordered proteins demonstrate a maximum frequency between 1644 and 1655 cm^−1^, whereas pure ß‐sheet structures show a maximum frequency at 1632 cm^−1^. The assay determines the secondary structure distribution of the antibody extracted biomarker such as TDP‐43, which is indicative for the misfolding state of the total fraction. The higher the proportion of misfolded TDP‐43 is, the lower is the recorded amide I maximum frequency directly indicating the grade of misfolding. This methodology, procedural steps for sensor functionalization, and procedures for data analyses were previously reported in detail for Aß^6^, ^7^ and Tau^8^ in CSF and/or plasma extracted from Alzheimer´s disease patients, other dementia and controls.

Briefly, highly specific and sensitive capture antibodies are covalently attached to the sensor surface, followed by surface inertization via casein blocking solution to avoid unspecific binding and to solely extract the all isoforms of the biomarker fraction from CSF. Unspecifically bound proteins are removed from the sensor surface by excessive rinsing with buffer. Thus, only the desired biomarker is detected by the immuno‐infrared sensor. For TDP‐43 extraction we used 10 µg/mL recombinant oligoclonal TDP‐43 antibody (Thermo Fisher Scientific, Waltham, Catalog# 711051) dissolved in phosphate buffer (50 mmol/L Na_2_HPO_4_/NaH_2_PO_4_, pH 8). It represents a mixture of monoclonal recombinant antibodies against different epitopes of TDP‐43 to facilitate the complete extraction of total TDP‐43 from CSF. The immunogen used for antibody production corresponds to human TARDBP (aa 130‐275). Specific binding of human TDP‐43 was further analyzed by siRNA mediated knockdown of TDP‐43 and antibody performance was evaluated by western blot using recombinant TDP‐43 as shown in the manufacturer´s description (Thermo Fisher Scientific, Waltham, Catalog# 711051). Instrumentation and data preprocessing comprising elimination of spectral contributions of water vapor were described previously in great detail and applied in the present study.^6^, ^9^

### Statistical analysis

A Grubbs test was carried out before the data analysis to exclude outliers, which, however, were not identified. A Shapiro–Wilk test was used as statistical significance test to evaluate the sensor read‐out values (distribution of the amide I maximum frequency) for normal distribution. Thus, nonparametric Mann–Whitney U‐tests were performed to calculate significant group differences between the three diagnostic groups. The chi‐squared test was used for a comparison of gender differences. Spearman rank correlation coefficient was used to examine correlations between clinical characteristics and TDP‐43 misfolding. To determine the decisive diagnostic threshold for group differentiation (listed in cm^−1^), receiver operating characteristic (ROC) analyses including calculation of the area under curve (AUC) were performed 5 times with different subsets comprising five ALS cases versus five PD subjects and their corresponding amide I maximum values and the same procedure with ALS versus Ctrl. Analyses revealed a mean threshold at 1643 cm^−1^, whereas lower values were indicative for TDP‐43 misfolding, which resulted in best discrimination accuracy with highest sensitivity and specificity. This threshold was set as discriminating threshold for analyzing the whole study group. Data were analyzed using the software Origin2019 by OriginLab (Software version 9.60).

## Results

We applied the immuno‐infrared sensor to determine the secondary structure distribution and therefore the overall misfolding state of TDP‐43 in CSF of 36 ALS cases, 30 PD cases and 24 Ctrl. All groups did not differ significantly in age (ALS 66 ± 10 years vs. PD 69 ± 13 years, *P* = 0.81; ALS vs. Others 65 ± 17 years, *P* = 0.54). The ALS female ratio (61%) versus PD (33%) was significantly different (*P* = 0.09), but there was no significant difference between ALS versus Ctrl (46%, *P* = 0.24). We identified a weak correlation of the CSF‐to‐serum albumin concentration quotients (Qalb) with the amide I maximum positions in ALS (*P* = 0.03, *r* = −0.36) and Ctrl (*P* = 0.03, *r* = −0.55), but not within the PD group (*P* = 0.18, *r* = −0.25). The average disease duration of ALS patients was 1.5 years and mean ALSFRS‐R score was 38 points at the time point of examination. 25 ALS patients had a spinal and 11 patients showed a bulbar disease onset (for details see Table 1).

**Figure 2:**
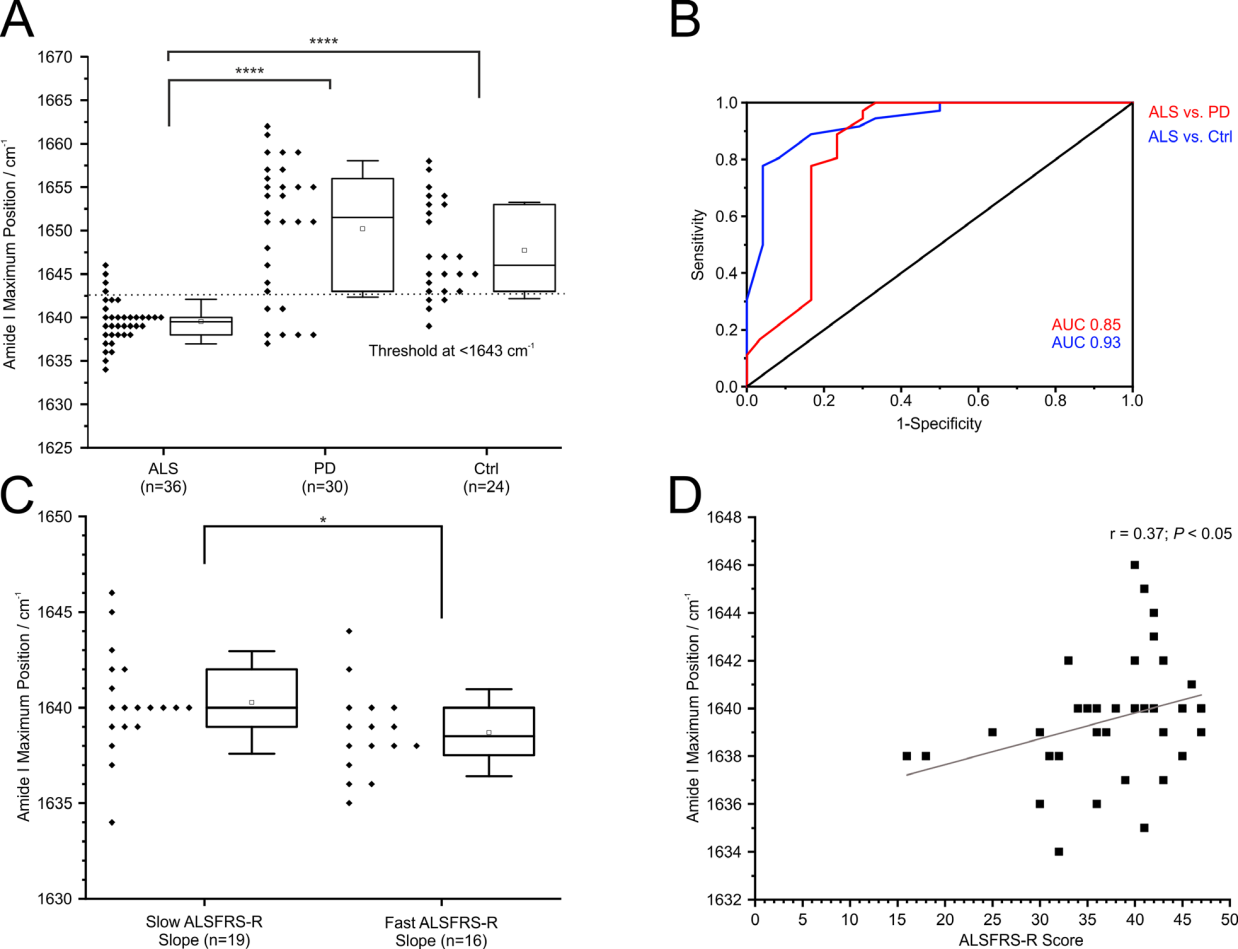
Immuno‐infrared sensor analysis of TDP‐43 secondary structure distribution in CSF. (A) Amide I band frequencies of patients with amyotrophic lateral sclerosis (ALS), Parkinson’s disease (PD), and other neurological disorders (Ctrl) (every diamond represents a single patient). A threshold of 1643 cm^−1^ (dotted line) discriminates ALS versus PD (sensitivity 89%, specificity 77%) and ALS versus Ctrl (sensitivity 89%, specificity 83%). Box and whisker plots show median (vertical line), mean (square), interquartile range (boxes), and standard deviation (whiskers). (B) ROC analyses revealed an AUC for ALS versus PD 0.85 (red) and for ALS versus Ctrl 0.93 (blue). (C) Amide I band frequencies of fast compared to slow progressing ALS patients according to ALSFRS‐R slope (cut‐off 0.5) (every diamond represents a single patient). (D) Spearman rank correlation between ALSFRS‐R score and the amide I band frequency. ****P < 0.0001; *P < 0.05.

A lower TDP‐43 amide I maximum frequency reflects a higher content of beta‐sheet enriched protein structures indicative of an increased amount of misfolded TDP‐43. In our study the amide I maximum frequency was significantly lower in CSF samples from ALS cases (1639.5 ± 2.6 cm^−1^ SD) compared to PD subjects (1651 ± 7.7 cm^−1^ SD) and Ctrl (1646 ± 5.5 cm^−1^ SD), suggesting misfolding of TDP‐43 in ALS cases (Fig. 2A, *P* < 0.0001). To determine the diagnostic potential of TDP‐43 misfolding for differentiation of ALS versus PD and ALS versus Ctrl, we performed a ROC analysis on amide I maximum data resulting in an AUC of 0.85 (95%‐CL 0.75–0.95) for ALS versus PD and an AUC of 0.93 (95%‐CL 0.87–0.99) for ALS versus Ctrl. The threshold at 1643 cm^−1^ revealed a sensitivity of 89 % and a specificity of 77 % for the comparison of ALS versus PD and 89% Sensitivity and 83% Specificity for ALS versus Ctrl (Fig. 2B). Moreover faster progressive ALS patients (*n* = 16) with ALSFRS‐R slope ≥ 0.5 points/month showed significantly lower amide I maximum frequencies compared to slower progressive patients (*n* = 19) with ALSFRS‐R slope < 0.5 points/month (*P* < 0.05) (Fig. 2C). Importantly, the analysis of the TDP‐43 measurements and clinical parameters revealed a positive correlation between the amide I maximum frequency and ALSFRS‐R scores (*P* = 0.03, *r* = 0.37) (Fig. 2D).

## Discussion

In this pilot study, we investigated whether the secondary structure distribution of TDP‐43 is a potential biomarker candidate for ALS. The presence of TDP‐43 protein in human CSF measured by standard immunoassays was already shown in different studies.^1^ Fibrillary structures consisting primarily of β‐sheet enriched protein species including TDP‐43 can be indicators of protein misfolding and lead to accumulation of aggregates as well as the formation of cellular deposits in several neurodegenerative diseases.^4^, ^13^, ^14^

Our study revealed a significant increase of TDP‐43 protein conversion to β‐sheet enriched structures in CSF samples from ALS cases as compared to PD and Ctrl. Tests for diagnostic accuracy using a simple discriminative threshold resulted in a sensitivity of 89 % and a specificity of 77 % for TDP‐43 misfolding in case of ALS versus PD and 89% and 83% for ALS versus Others. Furthermore, ALS patients with clinically faster progression and patients in more progressed disease stages showed higher β‐sheet content of TDP‐43. We observed weak correlation of the Qalb and amide I maximum position for ALS and Ctrl, but not for PD. We suggest no direct effect of elevated albumin levels on our measurements, because unspecific binding of albumin would rather lead to higher amide I frequencies, since it consists primarily of helical structures and western blot analyses excluded unspecific binding of albumin. Therefore, a higher number of patient examinations should clarify this point.

Moreover no specific binding of the antibody was achieved in CSF as evaluated by western blot. This was most likely due to the low levels of TDP‐43 in CSF, as shown in other studies.^15^, ^16^ Limitations of the study are the cross‐sectional design without longitudinal follow‐up and the limited number of samples. However, it comprised a multi‐center design of four academic study locations and included a group of controls with other neurological disorders. A correlation of the findings to other neurodegenerative biomarkers for ALS would enable a further analysis of its value as biomarker.

In conclusion, we present for the first time a TDP‐43 structure‐based biomarker for ALS, which should be validated in larger studies.

## Authors Contributions

L.T. and K.G. contributed to conceptualization and supervision. L.B., R.Gü., A.N., L.T., and K.G. contributed to methodology. L.B., R.Gü., A.N., L.T., and K.G. contributed to validation. L.B., R.Gü., and L.T. contributed to formal analysis. L.B., R.Gü., J.C.K., P.L., S.K., T.H., A.‐S.B., L.T., and K.G. contributed to investigation. L.B. and R.Gü. contributed to writing—original draft preparation. J.C.K., P.L., S.K., T.H., A.H., R. Go., A.N., L.T. and K.G. contributed to writing—review and editing. L. B. and L.T. contributed to project administration. All authors have read and agreed to the published version of the manuscript.

## Conflict of Interest

P.L and L.T. are inventors on a patent for the use of Fasudil for the treatment of Amyotrophic lateral sclerosis (EP 2825175 B1, US 9.980,972 B2). All other authors declare that there are no conflicts of interest.
